# Heavy Metal and Rice in Gluten-Free Diets: Are They a Risk?

**DOI:** 10.3390/nu15132975

**Published:** 2023-06-30

**Authors:** Karla A. Bascuñán, Claudia Orosteguí, Juan Manuel Rodríguez, Leda Roncoroni, Luisa Doneda, Luca Elli, Magdalena Araya

**Affiliations:** 1 Department of Nutrition, School of Medicine, University of Chile, Santiago 8380453, Chile; kbascunan@uchile.cl; 2 Institute of Nutrition and Food Technology, University of Chile, Santiago 7830490, Chile; corosteguism@gmail.com (C.O.); juan.rodriguez@inta.uchile.cl (J.M.R.); 3 Center for Prevention and Diagnosis of Celiac Disease, Gastroenterology and Endoscopy Unit, Fondazione IRCCS Ca’ Granda Ospedale Maggiore Policlinico, 20122 Milan, Italy; leda.roncoroni@unimi.it (L.R.); lucelli@yahoo.com (L.E.); 4 Department of Biomedical, Surgical and Dental Sciences, Università degli Studi di Milano, 20122 Milan, Italy; luisa.doneda@unimi.it

**Keywords:** celiac disease, gluten/wheat related disorders, gluten-free diet, rice, arsenic, copper, heavy metals

## Abstract

A gluten-free diet (GFD) is the treatment of choice for gluten-related disorders. It has been associated with macro- and micronutrient deficiencies. Recently, consumption of arsenic-contaminated rice has raised concern because of the potential greater risk that it may represent for people on GFDs, whose rice consumption is high, since it is a fundamental cereal in GFDs. We reviewed the data published over the last 20 years in Medline and Scielo, in English, French and Spanish, on four metals (As, Hg, Cd, and Pb), to assess whether the evidence suggests that celiac disease or consumption of a GFD is associated with increased levels of blood/urinary metal concentrations. The review revealed a few articles that were directly related to the four metals and their relationships with a GFD. The evidence supports that rice-based products are a relevant source of As and other metals. Clinical studies and evaluations based on NHANES have indicated that persons on GFDs have higher As and Hg blood/urinary levels, suggesting that the diet and not the disease is responsible for it. The levels described are statistically significant compared to those of persons on complete diets, but far from toxic levels. The question of whether higher exposure to heavy metals associated with a GFD is biologically relevant remains unanswered and deserves study.

## 1. Introduction

A gluten-free diet (GFD) is the treatment of choice for patients with celiac disease (CD) and gluten-related disorders (GRDs) [1]. A GFD eliminates natural foods/ingredients containing gluten and requires modifying processed food production to avoid gluten contamination. As a result, gluten-free products are often higher in fat, sugar, and energy content [2,3,4,5]; lower in magnesium, iron, zinc, and folate; and frequently, are poor sources of protein, fiber, iron, and B vitamins [6,7], as compared to their gluten-containing counterparts. Because of its low cost, rice is one of the most common alternatives used to replace wheat, rye, and barley in gluten-free foods.

Historically, the main health concern raised when dealing with gluten-free foods has been the potential aforementioned nutrition deficiencies. However, in recent years, concerns regarding heavy metals contamination in patients following GFDs has appeared to be a new scenario, because rice-based cereals and baby foods rich in rice have been found to contain high concentrations of arsenic (As) [8,9,10]. This has led to establishing specific limits for inorganic arsenic content in foods, including lowering the accepted As content in baby foods by half [11,12]. The European Commission (EC) modified the limit to 0.1-mg/kg for inorganic arsenic in rice food products for infants and young children. Limits also included non-parboiled milled rice (polished or white rice) (0.20 mg/kg limit); parboiled rice and husked rice (0.25 mg/kg); and rice waffles, wafers, crackers, and cakes (0.30 mg/kg limit for [11]. In 2016, the U.S. FDA proposed a limit, or “action level,” of 100 ppb for inorganic arsenic in infant rice cereal [10]. 

**Figure 1:**
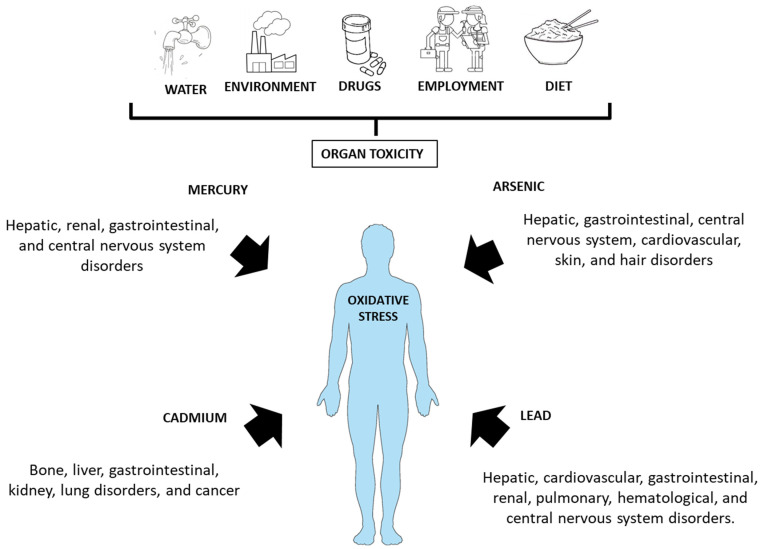
Environmental factors that may contaminate the diet and have toxic effects on human health.

Rice (*Oryza sativa* L.) accumulates arsenic, with concentrations up to ten times higher than other cereals such as wheat. Rice grown under flooded conditions favors arsenic solubility in the soil and uptake into the plant, and therefore it is a major dietary source of arsenic for populations that consume relatively low drinking water concentrations of As [9]. Today, there is increased awareness of the health risk posed to humans by arsenic-contaminated rice consumption and it is a recognized threat to food safety [13]. The possibility of higher exposure to As through rice-rich processed foods, indeed, raises a problem because, although there are no studies that have exactly measuring it, daily practice clearly indicates that celiac patients and gluten/wheat intolerant persons consume much more rice than people on regular, non-restrictive diets [14]. Relevant metal contamination in farmland soil is a known threat to food security because of the potential bioaccumulation of heavy metals in crops such as rice, corn, and other vegetables grown in contaminated soil [15]. The development of the economy, improper disposal of wastewater and solid waste by humans, and the use of chemical fertilizers and pesticides have resulted in pollutants entering farmland soils through different routes, causing paddy field pollution [16,17,18] (Figure 1). The relationship between these phenomena and a GFD is unclear but relevant. Recent meta-analyses [19] have suggested that higher consumption of white rice may increase the risk of developing type 2 diabetes, which is a condition closely related to celiac disease; the positive association between white rice intake and incidence of diabetes has been reported to be stronger among Japanese (*n* = 59,288) [20] and Chinese cohorts (*n* = 64,227) [21], two groups that are known to maintain high rice consumption.

We conducted a preliminary assessment by searching for information on GFD, rice, and metals and found that the topic has been barely investigated; only six studies were identified. Therefore, we conducted an extensive literature review by searching for articles related to this topic in a wider sense, published during the last 20 years in Medline and Scielo, using the terms gluten-free diet + metals + free full text + observational study + humans + English, French, and Spanish, on the contents of four metals (As, Hg, Cd and Pb) and their relationships to diet. The objective of the review was to assess whether the evidence suggested that CD or consumption of a GFD, accepting the assumption that a GFD diet promotes high rice intake, was associated with increased levels of blood/urinary metal concentrations, and whether these, at physiological, supraphysiological, or toxic levels, represented a potential risk for human health. Most research on rice and human health has been conducted in Asia, with no reference to CD [22]. The results of our literature review present the total number of published data found and are presented as follows: GFD and treatment of CD and GRDs; As, Pb, and Cd bioaccumulation in persons on GFDs; metals in gluten-containing and gluten-free foods; rice cultivars and As in contaminated soils; future perspective and final comments.

## 2. Gluten-Free Diet and Treatment of CD and GRDs

Celiac disease is one of the most common autoimmune gastrointestinal diseases; over the last decades, its prevalence indicates a mean annual increase in frequency currently calculated at 7.5% per year [23]. CD is triggered by gluten present in the diet and the disease involves autoimmune and inflammatory damage to the small intestine in genetically susceptible individuals. To develop celiac disease a person must inherit the genetic predisposition; however, about one third of the population carries the risk genes and only ~1% of the population develops the disease, indicating that genetics is not sufficient to explain the condition. The environment participates by providing the triggering factor, i.e., gluten, and the disease is activated by environmental factors which, until now, have not been fully understood, among which changes in eating habits and the intestinal microbiota are considered to be significant factors [24,25]; yet, current knowledge is insufficient to explain the mechanisms involved. Currently, the only treatment for CD is a GFD for life. 

Non-celiac wheat/gluten sensitivity. In recent years, an increasing number of persons in the general population have reported intestinal and extraintestinal symptoms after eating wheat. These patients have tested negative both for CD-specific serology and histopathology and for immunoglobulin E mediated assays; however, their symptoms improve on a GFD. This condition was formally described less than 10 years ago and, to date, it is not certain to what extent other wheat components, in addition to gluten [26], such as wheat amylase trypsin inhibitors [27] or wheat fructans (low-fermentable, poorly absorbed, short-chain carbohydrates or FODMAPs) [28] may contribute to the symptoms. Few reports refer to its prevalence, some estimates being at 4–6% of the population [29]. Patients suffering non-celiac wheat/gluten sensitivity are also treated with a GFD.

Wheat allergy. This immune adverse reaction is mostly mediated by IgE and is triggered by proteins contained in wheat and not necessarily in rye or barley, although IgE cross-reactivity has been described in some patients [30]. Treatment consists of avoiding eating any form of wheat, but gluten might be tolerated if it originated from non-wheat sources. The frequency of wheat allergy is largely unknown, although, recently, it was described at 3.9% in a series of 1203 adults [29]. In the USA, wheat is one of the eight most common foods to which people present allergic reactions. Regarding non-celiac wheat sensitivity, the only treatment is a wheat-free diet [31]; however, since the market only offers gluten-free products as alternatives, all these patients end up following a GFD.

Currently, a GFD is the only effective treatment for CD and GRDs. It is formed by uncontaminated naturally gluten-free foods (fruits, vegetables, sea foods, fish meat, poultry, legumes, nuts, and milk and dairy products) and processed foods that eliminate gluten as an ingredient or additive and avoid contamination during processing and distribution [32]. Gluten-free foods usually contain a limited number of ingredients, and they lack the fortification of their gluten-containing counterparts [33]. Rice has always been one of the mainin gredients in gluten-free foods due to its good palatability and low cost, and only recently have new ingredients emerged in the gluten-free food industry, such as quinoa, amaranth and other cereals, and pseudo cereals; they have better nutritional characteristics, but their higher cost limits their massification. Thus, rice remains to be the most consumed ingredient among those that maintain a GFD [34]. Among persons on complete diets in the USA, it is estimated that they consume 1 cup of rice daily [35]. 

The fashionable trend of eating “gluten free”. Consumption of gluten-free products has significantly increased and it has become an alimentary habit in the general population. There are scientifically unfounded perceptions that avoidance of gluten can improve health and help to lose weight and/or that gluten can be toxic for humans, thus, encouraging medically unjustified adherence to a GFD. Current medical recommendations indicate that only patients diagnosed with CD, non-celiac wheat sensitivity, and wheat allergy should eliminate gluten from their diets. Moreover, the available evidence does not support the idea that gluten might have adverse effects on human physiology and considering the well described nutrition deficiencies that may occur when a GFD is not properly supervised (see below), it seems largely justified not to maintain a GFD without a diagnosis to justify it.

## 3. Bioaccumulation of Heavy Metal in Persons on a GFD

Despite the relevance of deciding whether these elements are at physiological, supraphysiological, or toxic levels in a GFD and gluten-free foods, the literature review revealed only a few articles that were directly related to these metals and their potential relationship with a GFD. Assessment of blood Hg levels in Italian celiac patients (10 at the time of diagnosis, 20 on a GFD for at least six months, and 20 apparently healthy non-celiac controls) showed that in blood Hg concentrations were significantly higher in patients on GFDs than in celiac patients at diagnosis and in controls (10.2 ± 6.7 µg/L vs. 2.4 ± 2.3 µg/L and 3.7 ± 2.7 µg/L, respectively) [36].

The NHANES databases have been analyzed by different authors. Data originated from 2009 to 2012 in individuals older than 18 years investigated cases at the time of diagnosis of CD, those already on GFDs, and persons on complete gluten-containing diets. Comparing some metal concentration levels in blood and urine showed that persons on a GFD (including celiac patients and persons that follow GFDs without a diagnosis justifying this) showed significantly higher Hg, Pb, and As concentration levels than groups on complete diets, but only the As levels were above recommendations [37]. Statistical significance persisted when only a non-celiac group on GFDs was compared to controls. Further analysis of the NHANES databases (2007–2012), evaluating the blood levels of Pb and Hg against the presence of celiac disease seropositivity, hypothesized that heavy metals might trigger celiac autoimmunity. Against the expected results, positive CD serology showed no association with metal blood levels in adults and was significantly associated with lower metal levels in children. The authors interpreted these results as probably due to the malabsorption syndrome that is usually present in childhood CD. An interesting study by Wunsche et al. [8] compared urine As concentrations reported in two studies (both studies based on NHANES data) that included persons following a GFD or a complete gluten containing diet; in the first study, the As concentration values were 15.15 μg As/L versus 8.38 μg As/L [37], respectively, while in the other study, the values were 12.1 μg As/L and 7.8 μg As/L, respectively [38]). In this comparative study, the results were above those accepted safe for As, but within the acceptable range for Hg, Cd, and Pb. An additional assessment of the National Health and Nutrition Examination Survey (NHANES) 2003–2016 evaluated associations between rice consumption and arsenic metabolism, and between arsenic metabolism and insulin resistance in non-diabetic adults; the results suggested that rice consumption may contribute to lower monomethylarsonate (MMA%) that was further associated with higher insulin resistance, especially in obese individual [39].

Using a different approach, in Poland, Cd and Pb concentration levels were assessed in the deciduous teeth of 30 children with CD, 60 children with food allergies, and 60 healthy controls. Significantly higher Pb and Cd concentration levels were found in teeth obtained from celiac children as compared to the controls and to the food allergy group, with no differences between the controls and allergic children [40]. It is relevant that the greater Pb/Ca and Cd/Ca ratios among the celiac and food allergy groups vs. controls suggest that these two metals displaced other minerals in teeth. The literature search revealed only one case report that described a 39-year-old woman, diagnosed with CD 8 years before, who was admitted with symptoms of fatigue, diarrhea, dry mouth, anorexia, and memory loss. She was on a GFD with high rice and maize consumption. A 24 h urine analysis revealed 682.77 µg of As/g of creatinine. Symptoms remitted after three-day dimercaptosuccínic administration. Although undemonstrated, the suspected diagnosis was As intoxication due to high intake of rice-based gluten-free foods. 

## 4. Metals in Gluten-Containing and Gluten-Free Foods

Another approach to address these questions is by reviewing data on metal contents in different foods and soils. An assessment of 67 essential micronutrient and toxic trace element (As, Cd, Hg, and Pb) concentrations in gluten-containing and gluten-free foods [14] showed that the As contents in integral rice, rice flour, and gluten-free foods based on rice were higher than in flours and processed foods based on various other grains. Inorganic As in rice represented 63% of the total As measured in the tested products, and was highest in integral rice and enriched white rice. In general, rice and rice-based products contained significantly more As, Hg, and Pb and less Se, Fe, Cu, and Zn than their gluten-containing counterparts based on wheat [14].

## 5. Rice Cultivars and As in Contaminated Soils

Arsenic ranks 20th among the most abundant elements in the Earth’s cortex and it is one of the most toxic metalloids found in nature [41] (Figure 1). Speciation of arsenic depends on a variety of factors (chemical, physical, and biological), and its distribution and contamination on Earth are uneven, deriving from both natural (about one third) and anthropogenic processes (mainly industries and mine activities) [42,43,44]. It has been reported that parboiled and brown rice are more contaminated than basmati rice [45]. Long exposures to higher concentrations of As is considered to be a global problem because inorganic As that is present in contaminated ground waters has toxic effects and can cause serious damage to human health [46,47,48]. Contaminated ground waters have been described in India, Bangladesh, China, Vietnam, Nicaragua, Brazil, France, USA, Chile, and several other countries [49]. The main sources of As are drinking water, cultivars exposed to contaminated waters, and foods prepared with contaminated water [45,48,50]. Thus, for the world’s population, rice consumption is a major source of inorganic arsenic (As), a non-threshold class 1 carcinogen. 

## 6. Future Perspectives

To address the problems discussed in the previous paragraphs, the difficulties caused by dietary gluten content and rice safety must be solved. Here, we discuss two interesting lines of thought.

The diet. A well-known problem faced by the population today is the high consumption of processed foods that are poor in micronutrients and high in calories, sugars, and salt; this problem is clearly shared by those who are required to maintain a strict gluten-free diet. It is in this context that the risk of higher intake of heavy metals must be added, and therefore, there is a need to take a new look at the treatment of gluten-related disorders, for which a gluten-free diet is the only effective treatment. Important advances in the food industry have resulted in the development of a variety of ultra-processed gluten-free products, which have tremendously increased the variety of foods available to these patients. These foods look and taste good [33], but unfortunately, they have transformed the concept of a gluten-free diet (as a treatment) into a massive business, where the ingredients create a food [51], and the low cost of rice is a potent advantage to promote its use in the gluten-free industry [52], disregarding the fact that its higher As content can be transferred to whoever consumes it [53]. This is the basis for the proposal of incorporating the concept of “intelligent nutrition”, approaching a gluten-free diet as comprised mainly of naturally gluten-free foods; accepting this almost instantly transforms a restrictive diet into a healthy diet [54]. Unlike decades ago, currently, we recognize the high prevalence of overweight and obesity among celiac patients, which, in addition to a gluten-free diet rich in ultra-processed foods, adds other health risks to this group of patients, including problems of nutritional origin that clearly can be avoided [55]. Therefore, intelligent nutrition in a gluten-free diet includes two basic concepts. The first concept is related to gluten; the final product must be safe, i.e., without gluten coming from wheat, rye, and barley. To substitute it, different cereals can be used as raw materials, knowing that their nutritional content is better, such as pseudo cereals that have excellent protein content, among other qualities [56]. The second concept is that foods other than cereals should be preferred, the diet can be completed by increasing the contents of fruits, vegetables, legumes, cheeses, dairy products, among many others, and this will transform treatment into a healthy, varied, and appetizing diet [54].

The rice. Interestingly, in a paper by Mawia et al. [41], the authors discussed the possibility that by using new genome editing techniques (such as CRISPR/Cas-9) and base editing it was possible to develop novel rice varieties that were better suited for cultivation in As-polluted soils, with lower arsenic accumulation and without jeopardizing agronomic performance. In addition, the coupling of nanoparticles with phytochemicals is an emerging area in the field of phytonanotechnology. Through phytoengineering technology, future research should identify and isolate plant-derived bioactive compounds that show arsenic chelation effects and couple these compounds with nanoparticles to make phytonanoparticle compounds. These compounds could then be applied in As-contaminated paddy soils. With genetic engineering today, it is possible to develop rice cultivars with low As [57]. Additionally, application of modern agronomic practices such as supplementation of soils with specific mineral nutrients, for example, Fe, S, P, and Si, and civil engineering methods such as metal oxide nanoparticles have created favorable conditions for As precipitate formation, thus decreasing the bioavailability of As uptake by rice plants [58,59]. The adoption and planting of genetically modified rice cultivars with low As accumulation potential in soils supplemented with these minerals or metal oxide nanoparticles could offer a double advantage of reducing As accumulation in rice grain in the future.

## 7. Comments

This review shows that the controversy regarding heavy metal exposure and a GFD remains unsolved. The facts are that human beings are exposed to metals in various ways through drinking water and foods, and the evidence supports that rice-based products are a relevant potential source of such metals. Therefore, it is reasonable to think that high consumption of gluten-free products that frequently replace wheat by rice, may be a health risk. Direct evidence is scarce, it comes mainly from a study that reported increased Hg blood concentrations in celiac patients on GFDs [36] and another study that showed that the urinary concentration of As was significantly associated with rice consumption. Integral rice, rice flour, and gluten-free products based on rice are positively correlated with a GFD, but published research is limited, providing insufficient and challenging data. It is interesting that the higher As and Hg levels described in persons on GFDs, including not only celiac patients but any person on this diet, clearly suggest that the diet and not the disease is responsible for the results observed. The data based on the NHANES studies show that a GFD is associated with increased exposure to some toxic metals, but the exposure appears below toxic levels. In the case of As, the data support that As content in rice is higher than in other grains such as wheat, barley, and rye. Certainly, patients with GRDs greatly benefit from a GFD, provided they control the nutrition deficiencies potentially associated with it. Nevertheless, adhering to a GFD is challenging because it is difficult to strictly avoid gluten in processed foods, it causes psychological burden, and it diminishes nutritional health especially in relation to iron, calcium, thiamine, riboflavin, and folate.

On the one hand, heavy metal exposure, particularly Hg and Pb, may induce severe complications (abdominal colic pain, bloody diarrhea, and kidney failure, among others) when ingested in high doses [60]. On the other hand, low-dose exposure is a subtle and hidden threat, which after chronic, repeated intake may manifest by its complications, e.g., neuropsychiatric disorders including fatigue, anxiety, and detrimental impacts on intelligence quotient (IQ) and intellectual function in children [61]. In the case of a GFD, the results described may be considered minor increases, but they might be relevant bearing in mind that a GFD is usually lifelong. 

Rice is grown in flooded fields, where arsenic is found naturally in the soil and groundwater [62], and rice-based foods have shown high contents of total and inorganic arsenic and arsenic content seems to be correlated with the rice percentage present in rice-based foods [63]. Data on rice indicate that As locates especially in bran rice and is affected by growing conditions, which may result in lessening or increasing the risk of rice consumption; indeed, these issues deserve further study in humans [64]. Although without a confirmed diagnosis, the case report of a woman following a diet based mainly on rice, whose clinical condition rapidly remitted after treating the patient as As poisoning, also deserves attention [65]. Finally, the fact that the concentration levels described in persons on GFDs are statistically significant compared to those of persons on complete diets, but far from toxic levels, clearly maintains unanswered the question of whether higher exposure to heavy metals associated with a GFD is biologically relevant. 

**Figure 2:**
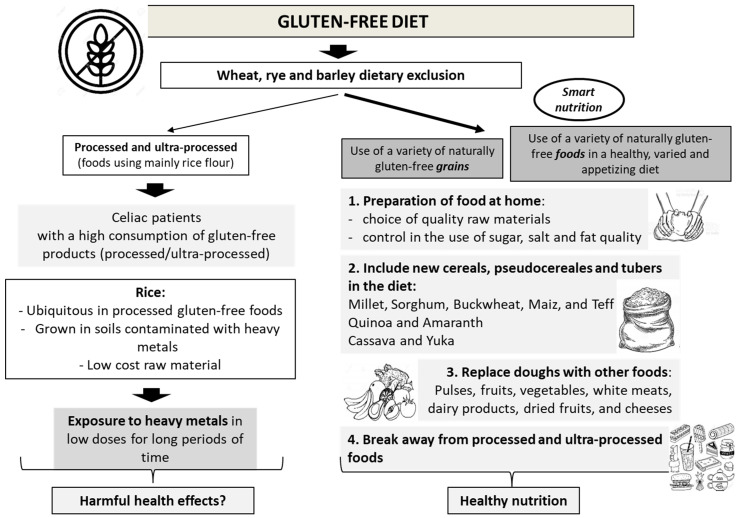
Importance of food choices (cereals or gluten-free foods) to maintain a healthy, diverse, and appetizing diet avoiding the intake of heavy metals that may threaten health.

Considering that a GFD is the only effective treatment for GRDs and until these issues are clarified, educational and professional support for patients on this diet should be promoted and improved. The person responsible for patient follow-up must be a professional trained in this type of diet who supervises that the diet is safe (gluten-free), varied in food, and sufficient in nutrients. The gluten-free Mediterranean diet [54] provides healthy and varied alternatives in terms of the use of cereals that can be found in many countries; it proposes a reduction in ultra-processed foods, favoring home-made preparations, using different cereals, many of which are not considered in most gluten-free products. Reducing consumption of processed foods (potentially high in heavy metals) would naturally increase the variety of naturally gluten-free cereals including teff, sorghum, maize, oats, and pseudo cereals such as buckwheat, amaranth, quinoa, and chia [54] (Figure 2), expanding the variety of flavors, textures, and nutrient contents and, indeed, improving the diet’s nutritional quality. 

## Data Availability

Not applicable.
